# Brain Age Gap as a Predictor of Early Treatment Response and Functional Outcomes in First-Episode Schizophrenia: A Longitudinal Study: L'écart d'âge cérébral comme prédicteur de la réponse en début de traitement et des résultats fonctionnels dans un premier épisode de schizophrénie : une étude longitudinale

**DOI:** 10.1177/07067437241293981

**Published:** 2024-11-10

**Authors:** Lejia Fan, Zhenmei Zhang, Xiaoqian Ma, Liangbing Liang, Liu Yuan, Lijun Ouyang, Yujue Wang, Zongchang Li, Xiaogang Chen, Ying He, Lena Palaniyappan

**Affiliations:** 1 Department of Psychiatry, National Clinical Research Center for Mental Disorders, and National Center for Mental Disorders, The Second Xiangya Hospital of Central South University, Changsha, China; 2 Douglas Mental Health University Institute, Department of Psychiatry, McGill University, Montreal, QC, Canada; 3 Robarts Research Institute, Schulich School of Medicine and Dentistry, Western University, London, ON, Canada

**Keywords:** treatment response, first-episode schizophrenia, brain age gap, global assessment of functioning, réponse au traitement, premier épisode de schizophrénie, âge du cerveau, fonctionnement global

## Abstract

**Objectives:**

Accelerated brain aging, i.e., the age-related structural changes in the brain appearing earlier than expected from one's chronological age, is a feature that is now well established in schizophrenia. Often interpreted as a feature of a progressive pathophysiological process that typifies schizophrenia, its prognostic relevance is still unclear. We investigate its role in response to antipsychotic treatment in first-episode schizophrenia.

**Methods:**

We recruited 49 drug-naive patients with schizophrenia who were then treated with risperidone at a standard dose range of 2–6 mg/day. We followed them up for 3 months to categorize their response status. We acquired T1-weighted anatomical images and used the XGboost method to evaluate individual brain age. The brain age gap (BAG) is the difference between the predicted brain age and chronological age.

**Results:**

Patients with FES had more pronounced BAG compared to healthy subjects, and this difference was primarily driven by those who did not respond adequately after 12 weeks of treatment. BAG did not worsen significantly over the 12-week period, indicating a lack of prominent brain-ageing effect induced by the early antipsychotic exposure per se. However, highly symptomatic patients had a more prominent increase in BAG, while patients with higher BAG when initiating treatment later showed lower gains in global functioning upon treatment, highlighting the prognostic value of BAG measures in FES.

**Conclusions:**

Accelerated brain aging is a feature of first-episode schizophrenia that is more likely to be seen among those who will not respond adequately to first-line antipsychotic use. Given that early poor response indicates later treatment resistance, measuring BAG using structural MRI in the first 12 weeks of treatment initiation may provide useful prognostic information when considering second-line treatments in schizophrenia.

## Résumé

**Objectifs:**

Le vieillissement accéléré du cerveau, c'est-à-dire les modifications structurelles du cerveau liées à l'âge et apparaissant plus tôt que prévu par rapport à l'âge chronologique, est une caractéristique désormais bien établie de la schizophrénie. Souvent interprétée comme une caractéristique d'un processus physiopathologique progressif qui est typique de la schizophrénie, sa pertinence pronostique n'est toujours pas claire. Nous étudions son rôle dans la réponse au traitement antipsychotique dans un premier épisode de schizophrénie.

**Méthodes:**

Nous avons recruté 49 patients schizophrènes n'ayant jamais été médicamentés, qui ont ensuite été traités par la rispéridone à une dose standard de 2 à 6 mg/jour. Nous les avons suivis pendant trois mois afin de catégoriser leur réponse au traitement. Nous avons obtenu des images anatomiques pondérées en T1 et utilisé le modèle XGboost pour évaluer l'âge du cerveau de chaque individu. L'écart d'âge cérébral est la différence entre l'âge prédit du cerveau et l'âge chronologique.

**Résultats:**

Les patients présentant un premier épisode de schizophrénie affichaient un écart d'âge cérébral plus prononcé que les sujets sains, et cette différence était principalement liée à ceux qui n'avaient pas répondu de manière adéquate après 12 semaines de traitement. L'écart d'âge cérébral ne s'est pas accru de manière significative au cours de la période de 12 semaines, ce qui indique l'absence d'effet de vieillissement cérébral induit par l'exposition précoce aux antipsychotiques à eux seuls. Cependant, les patients très symptomatiques présentaient une augmentation plus importante de l'écart d'âge cérébral, tandis que les patients dont l'écart d'âge cérébral était plus élevé au début du traitement présentaient par la suite des gains plus faibles sur le plan du fonctionnement global, ce qui souligne la valeur pronostique des mesures de l'écart d'âge cérébral pour les patients qui vivent un premier épisode psychotique.

**Conclusions:**

Le vieillissement accéléré du cerveau est une caractéristique du premier épisode de schizophrénie qui est plus susceptible d'être observée chez les personnes qui ne répondent pas de manière adéquate à l'utilisation d'antipsychotiques de première intention. Étant donné qu'une faible réponse en début de traitement indique une résistance future au traitement, la mesure de l'écart d'âge cérébral à l'aide de l'IRM structurelle pendant les 12 premières semaines de traitement peut fournir des informations pronostiques utiles lorsque vient le temps d'envisager des traitements de deuxième intention pour les patients atteints de schizophrénie.

## Introduction

Schizophrenia is an illness with a significantly high risk of premature mortality, with the average life expectancy of patients being reduced by about 15 years.^1–3^ Advanced cellular aging affecting multiple bodily systems, including the brain, is suspected to occur in schizophrenia.^4,5^ In fact, accelerated brain aging (ABA) i.e., the age-related structural changes in the brain appearing earlier than expected from one's chronological age, is well established in schizophrenia, even in the early stages of illness.^6–8^ The degree of ABA is quantified using the deviation of one's actual age from a prediction derived using multivariate structural neuroimaging features that relate to chronological age among healthy individuals (HC).^9,10^ T1-weighted structural imaging typically points towards an affected individual's brain being 0.4–7.8 years older than their chronological age for schizophrenia.^11–14^ Poor prognostic features such as longer illness duration^15^ and reduced gray matter tissue^16^ are associated with more pronounced ABA in schizophrenia and other psychiatric disorders.^10^ Schnack et al.^7^ found that the brain ageing in schizophrenia increased after schizophrenia onset, but slowed down five years later. These observations are often interpreted as evidence supporting a distinct early ‘progressive’ pathophysiology underlying ABA in schizophrenia.

There are two well-known challenges in arguing for a progressive pathophysiology (also called neurodegeneration or neuroprogression or brain tissue regression) in schizophrenia. One is the discord between clinical improvement seen in many subjects, especially after the first episode of schizophrenia (FES) and the longitudinal reductive changes reported in brain imaging studies.^17^ This has led to primary progressive pathophysiology being termed as a ‘myth’,^18,19^ and the brain age gap in schizophrenia being interpreted as a neurodevelopmental divergence.^13,20^ The second challenge stems from the possibility that several subgroups of patients with different primary pathophysiological trajectories may exist within the clinical phenotype of schizophrenia.^21^ While some subgroups may have a progressive pathophysiology, others may not.^22^ We do not have compelling indicators to identify if FES belongs to a putative ‘neuroprogressive’ subgroup at the outset. The presence of treatment resistance to first-line antipsychotics is associated with a higher propensity for structural changes in schizophrenia,^23,24^ Furthermore, a larger brain age gap is associated with lower global functioning among patients with established schizophrenia.^7,14,25^ These and other studies (29 studies as of 1 December 2023; see supplement 1 for an overview) indicate that a poor prognostic subgroup with higher functional impairment may exhibit a progressive pathophysiology, we cannot exclude the reverse causality; i.e., the effect that long-term, high dose antipsychotic exposure may have on the brain structure of patients with established treatment-resistance and poor functioning.^26^ Studying untreated FES in a prospective manner is crucial in this regard.

The emergence of later treatment resistance is often preceded by a lack of robust early response to antipsychotics in the first episode.^27–29^ Poor early response often leads to higher antipsychotic dose exposure.^30^ Thus, early poor response could indicate a propensity for putative neuroprogression reported in the treatment-resistant subgroup.^31^ Nonetheless, despite the associations reported between grey matter structure at baseline and later treatment response^32^ and the presence of much-pronounced brain aging in the presence of treatment resistance,^33^ no conclusive longitudinal link between accelerated structural changes and poor response has been established in FES. In the present study, we aim to investigate if (1) the brain age gap in schizophrenia is more pronounced in those who do not respond to the first-line antipsychotic treatment over 12 weeks, and (2) if poor responders show a progressive worsening of the brain age gap when treated with antipsychotics compared to responders. We also tested if the brain age gap before treatment onset predicts a lack of functional improvement over 12 weeks of first-line antipsychotic use. Our objective is to find out if using an antipsychotic medication without knowing how someone will respond later makes the underlying brain structure worse for those who are likely to be poor responders to the treatment. These answers will advance our current understanding of the determinants of treatment response.

## Methods

### Participants

**Figure 1.**
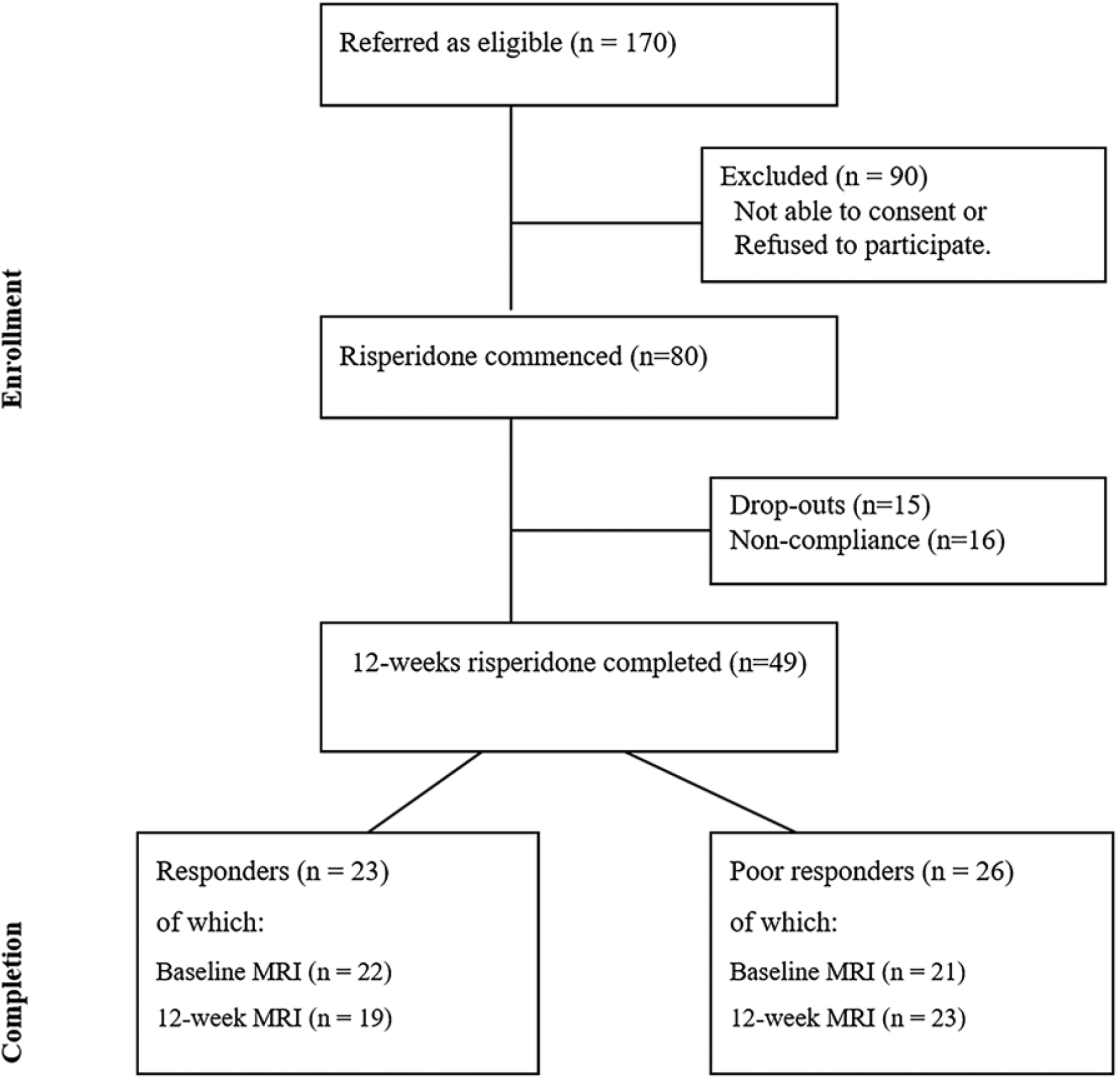
Flow diagram of first-episode schizophrenia FES: first-episode schizophrenia.

We identified 80 drug-naïve patients with first-episode schizophrenia (FES) who were about to begin treatment with risperidone from the Second Xiangya Hospital, and 67 HC recruited via community advertisements in the same geographical area in Changsha, Hunan, China between 2017 and 2022 and group-matched for age and sex. During this period, from hospital records we estimate approximately 4200 patients were seen at the respective clinics; of these only those who satisfied our inclusion criteria (drug naive at baseline, no substance use, no major medical illnesses or head injury, prescribed risperidone monotherapy by the treating clinician, able to give informed consent) were referred to the research team (*n* = 170); 80 FES provided informed consent and completed baseline scanning. Notably, the treatment was not assigned by the research team; but convenience samples of those who were about to start risperidone as monotherapy were recruited for the study. Diagnostic assessments for FES patients were completed by two experienced senior psychiatrists, based on the DSM-IV criteria.^34^ 16 were noncompliant with the treatment plan (8 FES stopped taking antipsychotics against medical advice, 8 received other interventions) and another 15 dropped out due to unwillingness to continue participating in this study for undisclosed reasons (Figure 1). In the end, 49 FES completed 12 weeks of risperidone at 2–6 mg/day (titrated as per clinical tolerance) and then were followed up. Psychotic symptoms and overall level of psychosocial, social and occupational functioning were assessed using the positive and negative syndrome scale (PANSS)^35^ and global assessment of functioning (GAF)^36^ respectively at baseline and 12 weeks. All HC had no current or past history of DSM-IV Axis I disorder themselves or a known family history of any mental disorder. All subjects are no concomitant medications were used at any time during the 12 weeks. Participants were excluded if they satisfied DSM-IV substance abuse or dependence criteria (except nicotine use) in the 12 months before treatment onset, had any known major physical illness, a history of prior antipsychotic exposure, or head injury, any contraindications for MRI.

All participants provided written informed consent to this study. As previously reported^37^ that there was no active treatment assignment, the study was deemed observational and not an interventional trial. The study procedures were registered in the Chinese Trial Registry (ChiCTR1800014844).

According on proposed criteria and working group consensus for remission in schizophrenia,^38^ responders are those who reach a rating of mild or less (equivalent to 1, 2, or 3) for all of the following items of the PANSS:^39^ delusions (P1), unusual conceptual organization (P2), hallucinatory behaviour (P3), blunted affect (N1), social withdrawal (N4), and lack of spontaneity (N6), mannerisms/posturing (G5), thought content (G9) after 12 weeks of treatment. We did not apply a duration criterion (stability) of remission, but only the severity criteria, i.e., clinically meaningful response based on symptomatic remission, in line with prior works.^39–41^ In FES, achieving cross-sectional remission has been shown to be a robust indicator of sustained remission over 6 months^42^ and 1 year.^43^

### MRI Data Acquisition and Processing

T1-weighted anatomical images were acquired within 2 days after enrollment and before starting the first dose of risperidone for FES (baseline scan), and 12 weeks (follow-up scan). HC were scanned only once, as treatment and response phenomena do not apply to unaffected individuals, and we did not expect a change in BAG over 12 weeks in HC.^44^ T1 image parameters, analysis of MRI data and quality control are detailed in the supplementary material.

### Brain age Prediction and age Correction

The brain age prediction models (one for females and one for males) were originally pretrained in a large database reported by Kaufmann and colleagues (45,615 individuals aged 3–96 years, of which a training sample for age prediction was based on 35,474, HC aged 3–89 years unaffected by the diagnostic conditions reported by the authors).^14^ The models extracted 1118 features from the HCP atlas^45,46^ including 360 cortical volume, 360 cortical surface area, and 360 cortical thickness (180 regions of interest from each hemisphere), alongside 30 subcortical volumes and 8 cortical summary variables. The models are based on gradient tree boosting using the XGboost method. The models’ code is available online (https://github.com/tobias-kaufmann/brainage) and executed in R (version 4.2.2). In line with the original study, we used separate models for males and females.

Brain age predictions have age-dependent bias; we used a well-established bias-adjustment procedure to account for this.^47^ This procedure fits a linear regression model between predicted age and chronological age of HC participants to obtain slope and intercept for evaluating offset value. Then, the age-corrected predicted brain age is computed for both HC and patients by subtracting an estimated offset value from their respective brain ages. The rationale for this method ensure that the model has a consistent error across the lifespan for HC.

We also applied SHapley Additive Explanations (SHAP) to calculate the contribution of each brain feature to brain age prediction (see supplement).

## Statistical Analysis

All statistical analysis was completed using SPSS 29. Demographic variables were compared across groups with the use of ANOVA and Chi-squared tests for continuous and dichotomous variables, respectively. All statistical analyses were conducted with age-corrected BAG values; unless specifically indicated, all references to BAG in the rest of this manuscript refer to corrected BAG measures. ANOVA was conducted to assess the effect of group with BAG or average BAG (mean of baseline and 12 weeks) as the dependent variable and the group as the independent variable, with education and sex as covariates and sought between-group differences post hoc using the least significant difference correction. A repeated measures ANOVA assessed the treatment effect with group (responders, poor responders) as the between-subject variable, time (baseline, 12 weeks) as a within-subject variable with education and sex included as covariates. The GAF improvement was predicted by baseline BAG using linear regression. Linear regression also determined relationships between the baseline symptom and the rate of change in BAG across all patients, with PANSS total score and subscales as candidate predictors respectively. Pearson correlation analysis assessed the relationship between maximal antipsychotic dosage (12 weeks), duration of psychosis and the rate of change in BAG across all patients. The Kruskal­–Wallis *H*-test and the Wilcoxon matched-pairs signed-rank test were used to assess group differences and time differences of SHAP values, respectively, due to the non-normal distribution of SHAP values (confirmed using the Shapiro­–Wilk test).

## Results

### Demographic, Clinical Features

Twenty-three patients were classified as responders and 26 were classified as poor responders after 12 weeks of continuous treatment. As shown in Table 1, in the final dataset, the three groups were matched in demographic characteristics, except for education level where both HC and responders were better educated than poor responders. Responders and poor responders did not differ in duration of psychosis and risperidone dosage at 12 weeks. Poor responders had a higher symptom burden and lower GAF scores compared to responders at both baseline and 12 weeks (as they did not achieve clinical remission despite an improvement in symptom profile compared to the baseline). Both responders and poor responders showed a statistically significant improvement in the overall symptom domains with no notable differences between groups. Responders had higher GAF improvement (*t* = −2.05, *p* = .048; *M*(*SD*): poor responder: 17.84(12.48), responder: 27.63(16.68)).

**Table 1.** Demographic, Clinical Features and Corrected BAG of Participants.

	Responders *N* = 23	Poor responders *N* = 26	Health controls *N* = 67	*F*/χ2/*t*	*p*
Age, years	22.35 ± 5.23	19.58 ± 5.49	20.67 ± 3.45	2.49	.087
Sex (male/female)	8/15	12/14	38/29	3.49	.174
Education level, years	12.74 ± 2.80	10.96 ± 3.13	13.84 ± 2.95	8.93	<.001
^a^Duration of psychosis, months	9.09 ± 9.63	14.62 ± 19.72	___	1.18	.246
Dose of risperidone at 12 weeks (mg/d)	4.08 ± 1.86	3.79 ± 1.03	___	0.45	.549
Symptom severity and psychosocial functioning at baseline					
PANSS total	77.17 ± 16.28	101.73 ± 17.91	___	5.03	<.001
PANSS positive	19.57 ± 5.26	25.81 ± 5.35	___	4.11	<.001
PANSS negative	18.22 ± 7.31	27.50 ± 8.80	___	4.03	<.001
PANSS general	39.39 ± 9.56	48.42 ± 9.67	___	3.28	.002
GAF	46.54 ± 10.18	38.80 ± 10.27	___	−2.59	.013
Symptom severity and psychosocial functioning at 12 weeks					
PANSS total	44.58 ± 5.95	66.00 ± 12.23	___	7.41	<.001
PANSS positive	9.05 ± 2.09	14.35 ± 4.16	___	5.34	<.001
PANSS negative	11.89 ± 3.43	21.35 ± 6.85	___	5.80	<.001
PANSS general	23.63 ± 3.37	30.30 ± 5.72	___	4.70	<.001
GAF	72.50 ± 12.04	55.68 ± 8.62	___	−5.00	<.001
Age-corrected BAG at baseline	2.06 ± 8.35	3.40 ± 9.04	0.001 ± 7.38	1.81	.170
Age-corrected BAG at 12 weeks	3.10±7.92	8.09 ± 7.77	0.001 ± 7.38	9.33	<.001

Data are presented as mean ± standard deviation unless otherwise specified.

^a^ Duration of psychosis is defined as the number of months between the onset of the first psychotic symptom and the date of diagnosis and treatment initiation (same day in all cases); GAF: global assessment of functioning.

As shown in Supplementary Table S1, patients who dropped out did not differ from those who were included in the study in the distribution of age, education, gender, and symptom burden.

### Comparison with Healthy Controls

We compared the age-corrected BAG values in the two patient groups with a sample of healthy controls, to estimate the degree of deviation from age and sex-matched healthy population. Patients with FES (as one group) had significantly higher BAG compared to HC at baseline (*F*_1,106 _= 4.68, *p* = .033, partial η^2^ = .042) (BAG: mean (*SD*) in HC = .001(7.38); FES = 2.71(8.62)) and 12 weeks (*F*_1,105 _= 13.64, *p* < .001, partial η^2^ = .115) (BAG: mean (*SD*) in HC = .001(7.38); FES = 5.83(8.14)).

**Figure 2.**
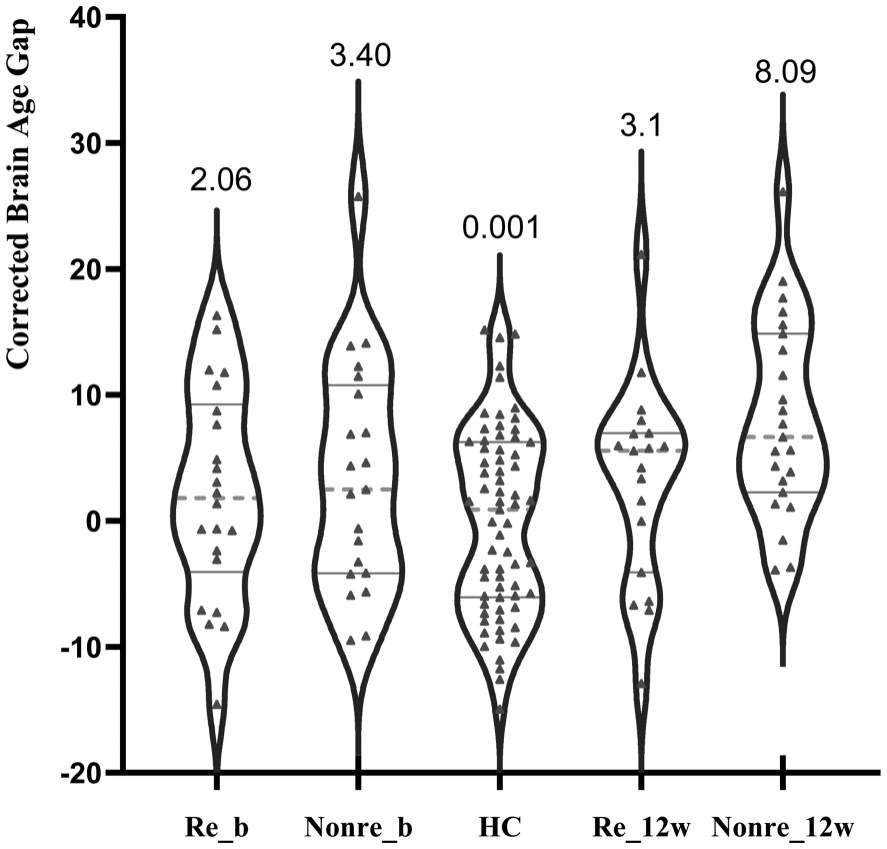
Age-corrected brain age gap at baseline and 12 weeks in patients classified as responders or poor responders at 12 weeks Re_b: responders at baseline; Poor_b: poor responders at baseline; Re_12w: responders at 12 weeks; Poor_12w: poor responders at 12 weeks; the graphs present the individual values, grey solid lines are first and third quartile, and the grey dotted line is median. The numbers are group mean.

As shown in Figure 2 and Table 1, when Responders were compared against healthy individuals, there were no differences at baseline or at 12 weeks. But poor responders had higher BAG compared to both Responders and healthy individuals at 12 weeks (*F*_2,104 _= 9.33, *p* < .001, partial η^2^ = .152), though not at the baseline (*F*_1,39 _= 1.81, *p* = .170, partial η^2^ = .033).

### Longitudinal Changes in BAG in Two FES Groups

**Figure 3.**
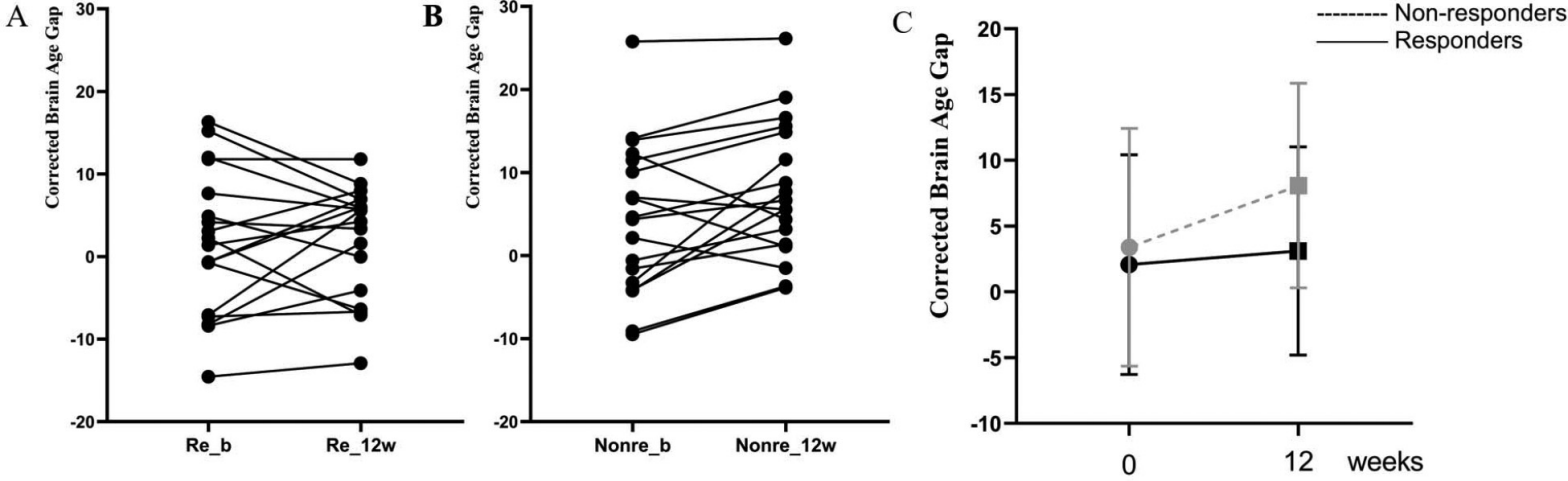
Age-corrected brain age gap at baseline and 12 weeks in patients classified as responders or poor responders at 12 weeks. (A) Individual trajectories across the 2 time points for responders; (B) Individual trajectories across the 2 time points for poor responders; (C) Group level changes in BAG; Re_b: responders at baseline; Poor_b: poor responders at baseline; Re_12w: responders at 12 weeks; Poor_12w: poor responders at 12 weeks.

A repeated measures ANOVA showed that there was a significant main effect of group (*F*_1,32 _= 5.32, *p* = .028, partial η^2^ = .143), poor responders had significantly higher average BAG (mean of baseline and 12 weeks) compared to Responders (average BAG: mean (*SD*) in poor responder =6.09(8.26); Responder =1.91(7.11)). Responders had an increase in BAG of 0.37 years (SD = 6.50) from baseline BAG of 2.06 years (*SD* = 8.35) to 3.10 years (*SD* = 7.92) at 12 weeks. Poor responders increased an average BAG of 3.25 years (*SD* = 5.71) from their baseline BAG of 3.40 years (*SD* = 9.04) to 8.09 years (*SD* = 7.77) at 12 weeks, resulting in a larger cross-sectional difference between the groups at 12 weeks. The group results and individual changes are shown in Figure 3.

There was no significant overall effect of time (*F*_1,32 _= 3.11, *p* = .087, partial η^2^ = .089), and there was no time*group effect on BAG indicating that the effect of response status on BAG is not dependent on the 12 weeks of antipsychotic exposure in this sample (*F*_1,32 _= 0.18, *p* = .672, partial η^2^ = .006).

### Baseline BAG and Overall Level of Functioning Improvement

**Figure 4.**
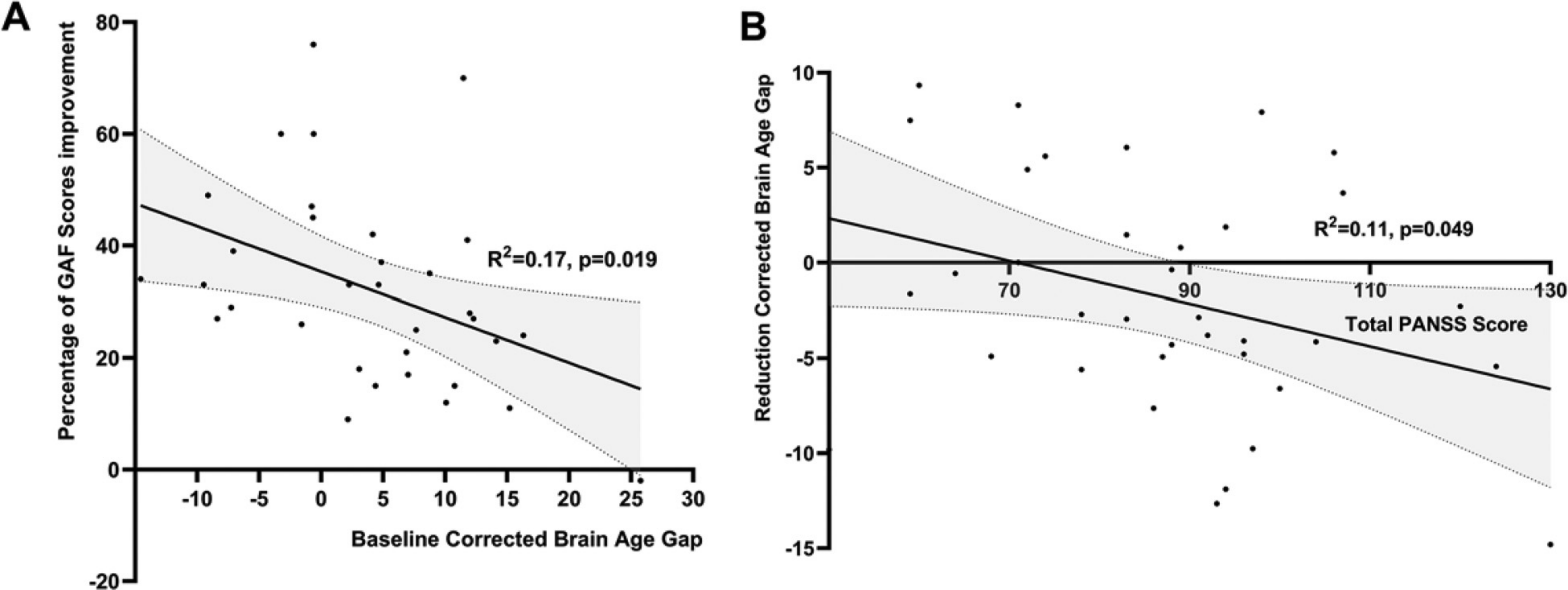
Relationship between age-corrected brain age gap and clinical features for all patients. (A) relationship between baseline BAG and percentage of GAF improvement; (B) relationship between total PANSS score at baseline and BAG change scores; GAF: global assessment of functioning; BAG: brain age gap. *R*^2^: coefficient of determination.

Regression analysis revealed that higher baseline BAG is related to lower percentage change in overall improvement in functioning for all patients (Beta = −.41; *t* = −2.47; *p* = .019), (figure 4), with correlation analysis indicating a significant negative relationship between baseline BAG and overall level of functioning improvement over time (*r* = −.3, *p* = .034).

### Factors Influencing Rate of Change in BAG Over 12 Weeks

Though poor responders had higher BAG at 12 weeks than responders, the rate of change in BAG did not differ significantly with responder status. We studied the effect of baseline symptoms, GAF scores, duration of untreated psychosis, and maximal antipsychotic dosage (at 12 weeks) on the rate of change in BAG across the patient group. As shown in Figure 4, a higher baseline total PANSS score was associated with a significant increase in BAG over time (Beta = −.33; *t* = −2.04; *p* = .049) and a lower baseline GAF score (Beta = −.40; *t* = −2.45; *p* = .020) were associated with a significant increase in BAG over time. None of the PANSS subscales at baseline correlated with BAG at baseline, 12 weeks or change scores (*p* = .072 to .228). We did not observe a significant relationship between the duration of untreated psychosis (*r* = .08, *p* = .633), the maximal dose of risperidone by 12 weeks (*r* = .08, *p* = .653) with the observed change in BAG over time.

We calculated 1118 features SHAP values for each subject to identify the brain features that contributed most to brain age predictions. We focus on the top 10 most relevant brain features based on the mean absolute SHAP value extracted from each group of different sexes. Group comparison of brain features contributing to the observed BAG (Shapley Additive Explanations) see supplement.

## Discussion

In this longitudinal study, we examined brain age in a cohort of patients with FES undergoing risperidone treatment for 12 weeks. We report two major findings: (1) Patients with FES who do not adequately respond to first-line risperidone therapy show an advanced brain age compared to responders. The patient-control difference in the brain-age gap was almost entirely driven by patients who were poor responders after 12 weeks of antipsychotic exposure. (2) The rate of change in BAG within the short 12-week window did not differ between responders and poor responders, but it was more pronounced in those with higher symptom burden at the baseline. Furthermore, larger BAG at treatment onset is related to a lower overall improvement in global functioning by 12 weeks, irrespective of the symptom remission status. Taken together, we demonstrate that the brain age gap is relevant to the early symptom remission and functional improvement trajectories in first-episode schizophrenia. The use of risperidone without any prior patient selection may result in insufficient symptom relief and functional gain for a subgroup with higher BAG (i.e., poor response to risperidone relates to higher BAG at baseline and poor GAF improvement over 12 weeks), but does not seem to worsen the accelerated brain ageing process per se (i.e., no time or time×response effect) in the first 12 weeks. These inferences must be considered in light of the various limitations of our study, as discussed below.

Our study has several strengths, including the recruitment of a fully antipsychotic-naive sample, with no cannabis use history, and a higher representation of women with FES (56% of patients), reducing a potential source of bias present in prior brain age studies,^48^ where only males were predominantly recruited.^49^ We also minimized drug-related variations by using a single antipsychotic medication. Several limitations are also worth considering when interpreting our results. First, we used XGBoost for BAG quantification given its recent success across various schizophrenia MRI datasets^16^; other algorithms may provide differing absolute values of BAG, although empirical data suggests that such variations are likely to be minimal.^50^ Furthermore, our inferences are limited to group comparisons and correlations, and all MRI scans were analyzed using the same BAG algorithm. Second, although we used a single antipsychotic, we did not fix the dose for every patient, and naturalistic titration based on tolerance and shared patient-clinician decision was allowed. We cannot rule out a misclassification error between the response-based groups as a result, but our method captured the real-world practice that clinicians employ when determining the degree of response. As substance use notably affects brain volume,^51^ excluding individuals with substance abuse/dependence improved the specificity for mapping BAG to psychosis in our sample. As more diverse samples are seen in the West where, unlike China,^52^ a higher prevalence of substance use is seen in first-episode clinics, caution is warranted when generalizing our findings. Though a causal role is not established, metabolic and inflammatory status may be other potential candidates that accelerate brain aging over a short period; but we lacked data to test these. Lastly, restricting our sample to those who were prescribed risperidone as monotherapy for 12 weeks limits the generalizability of our results to patients receiving other first-line antipsychotics such as olanzapine, and precludes interpreting poor response to this single agent as a defining feature of treatment resistance. Our results need replication with other antipsychotic medications.

Utilizing the brain age prediction models in our sample, we found that the mean BAG of HC was zero. This verified the validity of the normative model and our interpretations of patient-control differences, supporting the generalization of Kaufmann et al.'s^14^ brain age prediction models. The brain age-based approach is a global summary measure of deviations from age-expected structure. The absence of a time effect on BAG is consistent with other longer-term studies,^20,25^ there is no case to argue for a global neuroprogression in FES on the basis of our results. Higher BAG may instead result from early developmental deviations that affect the entire brain,^53^ or the set of features that emerged as the largest contributors in shapley additive explanations analysis (e.g., putamen, temporal regions, thalamus).^16^ Nevertheless, a more selective progressive pathophysiology in other individual regions (e.g., affecting pallidum upon treatment exposure as seen after low-dose risperidone use^54^) cannot be ruled out.^55^ A progressive process linked to biological aging may also occur during much later stages of schizophrenia (e.g., in midlife),^56^ especially in the context of metabolic aberrations.^57–59^

We noted an association between both symptom burden and poor functioning and BAG in patients. This is consistent with several prior cross-sectional studies on medicated patients (Supplementary Table S2^7,25,33^), and a prior study on untreated FES samples.^60^ We also demonstrate that BAG at baseline can provide meaningful information about early response and functional status upon treatment onset in a prospective manner. Of note, there was no significant worsening of BAG over the 12 weeks treatment period; thus the observed deficits do not occur as a result of treatment exposure,^11^ though we cannot rule out longer term, and higher dose antipsychotic exposure effects. Furthermore, for a substantial number of patients, the BAG values were within the range seen in healthy subjects; the observed case-control differences were attributable to the presence of a non-responder group among cases. Though HC were scanned only at a single time point, patients have larger BAG compared to HC both at the baseline and by 12 weeks. Thus, patients who have high symptom burden and poor early improvement are the most likely candidates with the putative pathophysiology linked to accelerated brain-aging.

## Conclusion

Higher brain age gap in schizophrenia is evident in the untreated first episode stage, predominantly affecting those who respond poorly to first-line treatment. Clinical utilization of this information requires replicating the prediction of treatment failures using brain age gap with first-line therapies other than risperidone. This will also allow us to study the likely real-world utility of the decision to consider second-line treatments such as clozapine, for example, by enriching response-based subtyping proposed elsewhere.^61^ When realized, this could become a promising application for structural neuroimaging in this illness.

## Supplemental Material

sj-docx-1-cpa-10.1177_07067437241293981 - Supplemental material for Brain Age Gap as a Predictor of Early Treatment Response and Functional Outcomes in First-Episode Schizophrenia: A Longitudinal Study: L'écart d'âge cérébral comme prédicteur de la réponse en début de traitement et des résultats fonctionnels dans un premier épisode de schizophrénie : une étude longitudinale Supplemental material, sj-docx-1-cpa-10.1177_07067437241293981 for Brain Age Gap as a Predictor of Early Treatment Response and Functional Outcomes in First-Episode Schizophrenia: A Longitudinal Study: L'écart d'âge cérébral comme prédicteur de la réponse en début de traitement et des résultats fonctionnels dans un premier épisode de schizophrénie : une étude longitudinale by Lejia Fan, Zhenmei Zhang, Xiaoqian Ma, Liangbing Liang, Liu Yuan, Lijun Ouyang, Yujue Wang, Zongchang Li, Xiaogang Chen, Ying He and Lena Palaniyappan in The Canadian Journal of Psychiatry
