# Supplementary material for: Brain Age Gap as a Predictor of Early Treatment Response and Functional Outcomes in First-Episode Schizophrenia: A Longitudinal Study: L'écart d'âge cérébral comme prédicteur de la réponse en début de traitement et des résultats fonctionnels dans un premier épisode de schizophrénie : une étude longitudinale
Source: Can J Psychiatry. 2024 Nov 10;70(3):240–50. doi: 10.1177/07067437241293981 (PMC11562934; doi:10.1177/07067437241293981)
Supplement: sj-docx-1-cpa-10.1177_07067437241293981 - Supplemental material for Brain Age Gap as a Predictor of Early Treatment Response and Functional Outcomes in First-Episode Schizophrenia: A Longitudinal Study: L'écart d'âge cérébral comme prédicteur de la réponse en début de traitement et des résultats fon [file sj-docx-1-cpa-10.1177_07067437241293981.docx]

**Brain Age Gap as a Predictor of Early Treatment Response and Functional Outcomes in First Episode Schizophrenia: A Longitudinal Study**

Lejia Fan MD^1,2^, Zhenmei Zhang MD^1^, Xiaoqian Ma MD PHD^1^, Liangbing Liang MD^3^, Liu Yuan MD PHD^1^, Lijun Ouyang MD^1^, Yujue Wang MD^1^, Zongchang Li MD PHD^1^, Xiaogang Chen MD PhD^1^, Ying He MD PHD^1+^, Lena Palaniyappan MD PHD^2,3+^

^1^Department of Psychiatry, National Clinical Research Center for Mental Disorders, and National Center for Mental Disorders, The Second Xiangya Hospital of Central South University, Changsha, China.

^2^ Douglas Mental Health University Institute, Department of Psychiatry, McGill University, Montreal, QC, Canada.

^3^ Robarts Research Institute, Schulich School of Medicine and Dentistry, Western University, London, ON, Canada.

+ Co-corresponding author

Corresponding Author:

Lena Palaniyappan: 6875 LaSalle Boulevard, Verdun QC Canda H4H 1R3 Email:[lena.palaniyappan@mcgill.ca](mailto:lena.palaniyappan@mcgill.ca)

Ying He: No. 139, Furong District, Changsha City, Hunan Province, China Email: yinghe@csu.edu.cn

**1. MRI data acquisition and processing**

T1 images were acquired using 3-dimensional magnetization-prepared fast gradient echo sequences for voxel tissue segmentation (TR = 2530 ms; TE = 2.33 ms; gap = 0.5 mm; flip angle = 7o; FOV = 256× 256 mm; number of excitations (NEX) = 1; slice thickness = 1.0 mm; and number of slices = 192). The obtained images underwent automated surface-based morphometry and subcortical segmentation using FreeSurfer (version 7.2.0; http://surfer.nmr.mgh.harvard.edu/). Preprocessing of these images included the removal of non-brain tissues as well as spatial and intensity normalizations. We used the multimodal Human Connectome Project (HCP) atlas^1^ for spatial labeling and extraction of the features including volume, area, and thickness measures. The segmentations and pial surface placement were checked in accordance with FreeSurfer recommendations. (<https://surfer.nmr.mgh.harvard.edu/fswiki/FsTutorial/TroubleshootingData>).

**2. SHapley Additive Explanations (SHAP) Values calculation**

Each feature of coefficient may indicate average contribution across sample in linear regression model, while in nonlinear tree-based model, each feature may have different contribution for each subject based on the individual prediction path took in the tree, so the model explanations should derive from participant level to elucidate how each individual prediction is made. SHAP employs game theory to calculate the marginal contribution of features^2^, which represents the extent of the model's change when a new feature is added. SHAP values were obtained utilizing the SHAPforxgboost package in R, accessible at（<https://cran.r-project.org/web/packages/SHAPforxgboost/index.html>）

**3. Demographic and clinical characteristics of dropouts and included FES**

Patients that dropped out did not differ from those who were included in the study in the distribution of age, education, gender, and symptom burden.

**Table S2. Demographic and clinical characteristics of dropouts and included FES**

|  | Dropouts FES (N=31) | Included FES (N=49) | t/χ2 | P |
| --- | --- | --- | --- | --- |
| Age | 22.65±6.53 | 20.96±5.45 | 1.25 | 0.216 |
| Sex (male/female) | 19/12 | 20/29 | 3.19 | 0.074 |
| Education | 11.63±3.39 | 11.96±2.96 | -0.45 | 0.654 |
| PANSS Total | 83.45±14.37 | 90.20±21.02 | -1.57 | 0.120 |

**4. Brain features contributing to the observed BAG (Shapley Additive Explanations)**

We calculated 1118 features SHAP values for each subject to identify the brain features that contributed most to brain age predictions. The top ten most relevant brain features based on mean absolute SHAP value were extracted from each group of different sex, and a union set was selected; thus, >10 predictors were theoretically possible in each group. A high degree of overlap was seen for most relevant brain features across groups, with 12 brain features making notable contributions as shown in figure S1. There were no significant group differences in the SHAP features (all p>0.05 after Bonferroni corrections for multiple testing). None of the SHAP values were significantly different between baseline and 12 weeks for non-responders or responders (p=0.102 to 0.983), indicating that the features predicting BAG were fairly constant across the groups and over time.


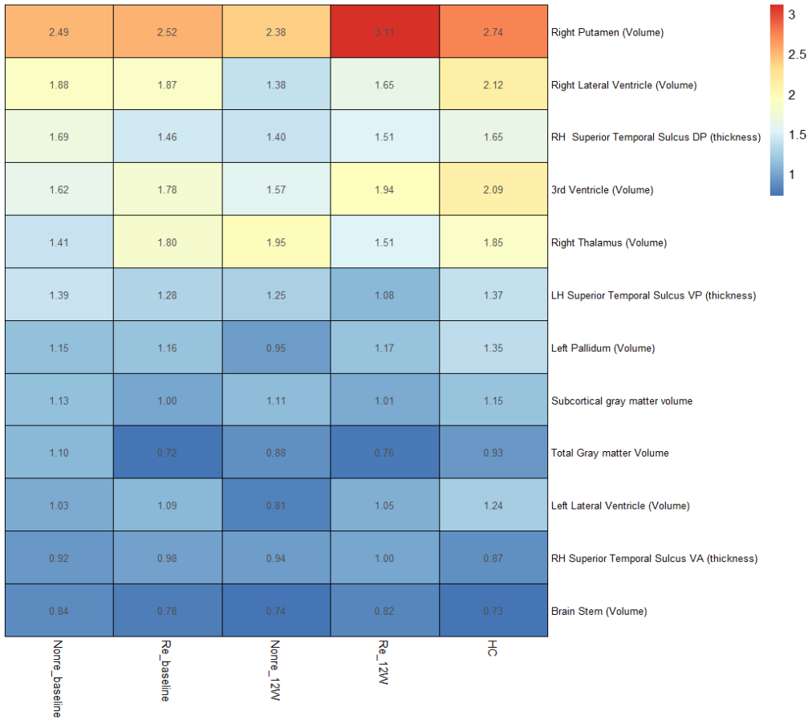


**Figure S1 SHAP values for most relevant brain features for brain age prediction** RH: right hemisphere; LH: left hemisphere; DP: dorsal posterior; VP: ventral posterior; VA: ventral anterior; Nonre_baseline: non-responders group at baseline; Re_baseline: responders group at baseline; Nonre_12W: non-responders group at 12 weeks; Re_12W: responders group at 12 weeks; HC: health controls.

**5. Overview of brain age prediction studies on schizophrenia**

**Table S2 Overview of brain age prediction studies on schizophrenia**

| **Study** | **Sample size** | **Subjects age range** | **Modality** | **Methods** | **Main results of BAG** | **Relationship between BAG and clinical variables** |
| --- | --- | --- | --- | --- | --- | --- |
| Kim et al. 2023^3^ | 214 HC  196 SCH (including 72 FES, 46 TRS) | 22-53 | T1 | ridge regression, support vector regression, and relevance vector regression | SCH>HC  TRS>FES>HC | 1.In SCZ, BAG was significantly positive associated with total and negative symptoms and CPZ equivalent.  2.In FES, BAG was significantly positive associated with executive function and CPZ equivalent.  3.In TRS, BAG was significantly positive associated with chlorpromazine equivalent. |
| Ballester et al. 2023^4^ | three public databases COBRE (90 SCH, 93 HC), MCIC (94 SCH, 109 HC), and UCLA (50SCH, 125 HC) and TOPSY (80 SCH, 34 HC) | 18-65 | T1 | XG boost | SCH >HC only in COBRE and MCIC dataset |  |
| Constantinides et al. 2023^5^ | 2803 SCH 2598 HC | 18-73 | T1 | multivariable ridge regression | SCH >HC | For SCH, BAG was not associated with specific clinical characteristics (age of onset, duration of illness, symptom severity, or antipsychotic use and dose) |
| Abram et al. 2023^6^ | 52 SCH 54 HC | 19-65 | T1 | Multivariate ridge regression | SCH=HC | For SCH, BAG was negatively correlating with Emotional well-being scores |
| Zhu et al. 2023^7^ | 194 SCH 330 HC | 20-80 | T1, DTI | machine learning (Regression Learner Tool） | 1.Brain volume and cortical thickness model: SCH> HC  3. Fractional anisotropy model: SCH=HC | No significant correlation was found between the brain age gap and PANSS any symptom and total score and chlorpromazine equivalent dosages for three model |
| Xi et al. 2022^8^ | Principal dataset: 60 SCH 60 HC  Replication dataset: 40SCH 40HC | 18-44 | DTI, two scans | Machine-learning | SCH>HC  SCH treatment after<baseline | 1.BAG was negatively associated with the positive score on PANSS in the principal data-set  2.BAG is negative correlation with semantic verbal fluency after medication for about 4 months. BAG was irrelevant to digit symbols, and forward and backward digit span.  3. the correlations were not significant between the changes in BAG and current antipsychotic dose and scan interval. |
| Chen et al. 2022^9^ | 147SCH 130 HC | 16-62 | T1, diffusion spectrum imaging | 10-fold cross-validation | SCH > HC in GM, WM, and multimodality models. | The BAG based on WM was positively associated with the negative symptom, and negatively associated with the intelligence quotient and onset age. |
| Demro et al. 2022^10^ | 163 psychotic disorders (105 schizophrenia, 17 schizoaffective disorder, 41 BP with psychotic features); 103 their first-degree biological relatives; 66 HC | 18-65 | T1, two scans | BARACUS model | Psychotic disorders > HC=relatives.  SCH=BP with psychotic features >HC  SCH>relatives  BP without psychotic> BP with psychotic | 1.Advanced brain-age was associated with lower cognitive and general functioning in the full sample. Among relatives, cognitive performance and schizotypal symptoms were related to brain-age gap.  2.Lower IQ, lower functioning, and female sex predicted greater brain-age gap in the full sample. |
| Haas et al. 2022^11^ | 84 early-stage schizophrenia  1169 HC | 16-37 | T1 | machine learning algorithm with a U-Net architecture | cognitively impaired patient subgroup>HC | 1.Totality of regional age-related changes was no any significant correlations with the PANSS positive or negative symptoms subscale scores, antipsychotic medication, MIRECC-GAF scores and WASI-II IQ within either cognitive cluster.  2.Voxel-level local brain-age was no significant association with current antipsychotic medication dose. |
| Huang et al. 2022^12^ | 138 SCH 205 HC | 20-60 | T1, resting, DTI | Multiple linear regression | SCH exhibited increased brain age in the youth group but not in the middle age group |  |
| Wang et al. 2021^13^ | 166 SCH 107 HC | 23-53 | DTI | machine learning | BAG was significantly elevated in the age >30 group in patients but not in age ≤ 30 group | BAG in patients was significantly and negatively associated with both working memory and processing speed. |
| Lee et al. 2021^14^ | ISMMS: 90 SCH 200 HC  COBRE: 76 SCH 87 HC | 11-52 | T1 | Machine learning six algorithms | SCH>HC for all algorithms |  |
| Man et al. 2021^15^ | 501 SCH 512 HC | 18-50 | T1 | support vector regression | SCH>HC |  |
| Lieslehto et al. 2021^16^ | 29 SCH 61HC | 20-43 | T1, two scans | machine learning | SCH: follow up >baseline  At baseline and follow up SCH>HC | Support vector machines decision scores had a positive relationship with the number of hospitalizations, disorder duration, and Chlorpromazine dose years |
| Rokicki et al. 2021^17^ | 750 HC and Alzheimer's disease (n = 54), mild (n = 90) and subjective cognitive impairment (n = 56), schizophrenia spectrum (n = 159) and bipolar disorder (n = 135) | 18-85.8 | T1/T2‐weighted ratio, arterial spin labelling | Random Forest | SCH>HC for almost all modalities |  |
| McWhinney et al. 2021^18^ | 183 First-episode psychosis 155 HC | Mean age 16.1 | T1, two scans | machine learning | 1.FEP>HC  2. For HC and FES: Baseline =follow-up | 1. For all subjects, greater baseline BMI predicted faster brain ageing; For each additional BMI point, the brain aged by an additional month per year；  2.Worsening of functioning (change GAF) over time and increases especially in negative and general symptoms on the PANSS were associated with greater annual rate of brain ageing in FEP.  3. for FEP, lower baseline functioning (GAF) and higher PANSS negative scores were associated with higher baseline BAG |
| Kuo et al., 2020^19^ | 26 SCH, 1009 HC, 30 MDD and 19 AD | 50-90 | T1, T2 | LASSO regression | SCH>HC  AD >HC | No significant correlation was found between with any clinical scales for any of the groups including HDRS, PANSS Positive scale, PANSS Negative scale, PANSS General Psychopathology scale, and PANSS Total scale. |
| Truelove-Hill et al., 2020^20^ | 43 SCH 53 HC | 16-22 | T1, resting | support vector machine | SCH>HC |  |
| Tønnesen et al., 2020^21^ | 648 SCH 185BD 990 HC | 17–68 | DTI | XGboost | SCH=BD>HC |  |
| Chen et al., 2020^22^ | 158 SCZ 60 HC | 22-71 | DTI | Cascade neural networks | SCH>HC in two model |  |
| Bashyam et al,2020^23^ | 448 HC (SCZ) 387 SCZ  204 HC (MDD) 204 MDD  513 HC(AD), 833 MCI, 353 AD | 3-95 | T1 | cross-validation | HC: −2.29 (4.707) (SCZ matched) SCZ: 0.75 (4.947)  HC: 0.11 (6.16) (MDD matched) MDD: 0.73 (5.57) |  |
| Shahab et al,2019^24^ | Sample 1: 81 SCZ, 53 BD, 91 HC  Sample 2: 67 SCZ, 80 HC | 18- 83 | T1, DWI | Random forest | 1.SCH>HC=BD  2. BD with psychosis = BD without psychosis |  |
| Kaufmann et al,2019^25^ | 1110 HC (SCZ), 1110 SCZ  459 HC (BD), 459 BD  208 HC (MDD), 208 MDD | 3-96 | T1 | XGboost | HC (SCZ): 0 (7.04), SCZ: 3.83 (7.98)  HC (BD): 0 (6.72), BD: 2.07 (7.39)  HC (MDD): 0 (6.28), MDD: 0.86 (6.53) | In SCH, larger BAG was associated with lower functioning (for example, insula brain age gap with GAF function) and with more negative symptoms (for example, temporal brain age gap with PANSS negative). |
| Hajek et al,2019^26^ | 43 HC 43 SCZ  60 HC 96 offspring BD (48 unaffected, 48 affected) | 15-55 | T1 | relevance vector regression | 1.SCH>HC  2.Unaffected offspring = Affected offspring | For FES, BAG was not associated with duration of illness or duration of untreated psychosis |
| Kolenic et al,2018^27^ | 120 FEP 114 HC | 18-35 | T1 | Relevance vector regression | 1.FEP>HC  2. Overweight > normal  3. previously medication naive participants > controls  previously medication naive FEP=medicated FEP | 1.BAG was positively related to BMI, and weight, not height;  2. BAG was not associated with cumulative exposure to antipsychotics;  3.Medication dosage at the time of scanning was not associated with BAG or BMI. |
| Chung et al,2018^28^ | 275 CHR individuals (including 39 who developed psychosis) and 109 HC  14FEP | 3-21 | T1 | penalized regression with L2 norms (ridge regression) | 1. CHR>HC  Younger: CHR_C>HC  2. FEP>HC  Younger: FEP> CHR_C>CHR_NC>HC | 1.baseline BAG did not significantly predict conversion to psychosis.  2.For CHR, mean GAF scores improved from baseline to follow-up when the brain age gap was within the mean absolute error range but did not improve for individuals with an underestimated or overestimated brain age gap. |
| Nenadić et al,2017^29^ | 45 SCZ 22 BD 70 HC | 21.4–64.9 | T1 | relevance vector regression | SCH>BD=HC |  |
| Schnack et al,2016^30^ | 341 SCZ 386 HC | 16-76 | T1, two scans | Support vector regression. | 1.progressively increased during follow-up (+1.24 years in addition to the baseline gap) for FES.  2. it decreased from 2.5 years/year just after illness onset to about the normal rate (1 year/year) approximately 5 years after illness onset for FES | 1.At follow-up, BAG was significantly negatively associated with GAF score and positively with antipsychotic dosage.  2.Brain age acceleration rate was negatively associated with GAF score and positively with PANSS total score at follow-up, and positively with number of hospitalizations, duration of hospitalization, and cumulative antipsychotic intake. The latter was also significantly associated with schizophrenia gap acceleration rate. |
| Koutsouleris et al,2014^31^ | 800 HC (train set)  141 SCZ 104 MDD  57 BPD  89 ARMS | 18-65 | T1 | Support vector regression | SZ=MD>AMRS>HC SZ>BPD>HC  RE-SZ>RO-SZ>AMRS-E=HC  RE-SZ>AMRS-L>AMRS-E=HC | 1.BAG predicted both patient status as well as negative and disorganized symptoms.  2. BAG was not associated with psychotropic medications, nicotine, and alcohol in the patient (SZ, MD, and BPD) and ARMS groups and no associations of sociodemographic parameters, nicotine/alcohol consumption, and different somatic conditions on BrainAGE in HCs. |

Note: SCZ: schizophrenia; HC: health controls; FES: first episode schizophrenia; TRS: treatment resistant schizophrenia; FEP: first episode psychosis; BP: bipolar disorder; MDD: major depression disorder; BPD: borderline personality disorder; ARMS: individuals in at-risk mental states for psychosis; ARMS-E: early at-risk mental states; ARMS-L: late at-risk mental states; RO-SZ: recent-onset schizophrenia patients; RE-S: recurrently ill schizophrenia patients. AD: Alzheimer disease; CHR_C: clinic high risk who developed psychosis; CHR_NC: clinic high risk who don't developed psychosis. BAG: brain age gap; DTI: diffusion magnetic resonance imaging; WM: white matter; GM: grey matter; GAF: global assessment of function.

**Reference**

1. Liang L, Silva AM, Jeon P, et al. Widespread cortical thinning, excessive glutamate and impaired linguistic functioning in schizophrenia: A cluster analytic approach. Frontiers in human neuroscience. 2022;16(954898.

2. Shapley L. Value for n-person games, contributions to the theory of games (Kuhn, HW, Tucker, AW Eds.). 307–317. Ann Math Stud. 1953;28(275-293.

3. Kim W-S, Heo D-W, Shen J, et al. Stage-specific brain aging in first-episode schizophrenia and treatment-resistant schizophrenia. International Journal of Neuropsychopharmacology. 2023;26(3):207-216.

4. Ballester PL, Suh JS, Ho NC, et al. Gray matter volume drives the brain age gap in schizophrenia: a SHAP study. Schizophrenia. 2023;9(1):3.

5. Constantinides C, Han LK, Alloza C, et al. Brain ageing in schizophrenia: evidence from 26 international cohorts via the ENIGMA Schizophrenia consortium. Molecular Psychiatry. 2023;28(3):1201-1209.

6. Abram SV, Roach BJ, Hua JP, et al. Advanced brain age correlates with greater rumination and less mindfulness in schizophrenia. NeuroImage: Clinical. 2023;37:103301.

7. Zhu J-D, Tsai S-J, Lin C-P, et al. Predicting aging trajectories of decline in brain volume, cortical thickness and fractional anisotropy in schizophrenia. Schizophrenia. 2023;9(1):1.

8. Xi Y-B, Wu X-S, Cui L-B, et al. Neuroimaging-based brain-age prediction of first-episode schizophrenia and the alteration of brain age after early medication. The British Journal of Psychiatry. 2022;220(6):339-346.

9. Chen C-L, Hwang TJ, Tung Y-H, et al. Detection of advanced brain aging in schizophrenia and its structural underpinning by using normative brain age metrics. NeuroImage: Clinical. 2022;34:103003.

10. Demro C, Shen C, Hendrickson TJ, et al. Advanced brain-age in psychotic psychopathology: evidence for transdiagnostic neurodevelopmental origins. Frontiers in Aging Neuroscience. 2022;14:872867.

11. Haas SS, Ge R, Sanford N, et al. Accelerated global and local brain aging differentiate cognitively impaired from cognitively spared patients with schizophrenia. Frontiers in psychiatry. 2022;13:913470.

12. Huang J, Ke P, Chen X, et al. Multimodal magnetic resonance imaging reveals aberrant brain age trajectory during youth in schizophrenia patients. Frontiers in aging neuroscience. 2022;14:823502.

13. Wang J, Kochunov P, Sampath H, et al. White matter brain aging in relationship to schizophrenia and its cognitive deficit. Schizophrenia research. 2021;230(9-16.

14. Lee WH, Antoniades M, Schnack HG, et al. Brain age prediction in schizophrenia: Does the choice of machine learning algorithm matter? Psychiatry Research: Neuroimaging. 2021;310:111270.

15. Man W, Ding H, Chai C, et al.: Brain age gap as a potential biomarker for schizophrenia: A multi-site structural MRI study. in 2021 43rd Annual International Conference of the IEEE Engineering in Medicine & Biology Society (EMBC) IEEE; 2021. pp. 4060-4063.

16. Lieslehto J, Jääskeläinen E, Kiviniemi V, et al. The progression of disorder-specific brain pattern expression in schizophrenia over 9 years. npj Schizophrenia. 2021;7(1):32.

17. Rokicki J, Wolfers T, Nordhøy W, et al. Multimodal imaging improves brain age prediction and reveals distinct abnormalities in patients with psychiatric and neurological disorders. Human brain mapping. 2021;42(6):1714-1726.

18. McWhinney S, Kolenic M, Franke K, et al. Obesity as a risk factor for accelerated brain ageing in first-episode psychosis—a longitudinal study. Schizophrenia Bulletin. 2021;47(6):1772-1781.

19. Kuo C-Y, Lee P-L, Hung S-C, et al. Large-scale structural covariance networks predict age in middle-to-late adulthood: a novel brain aging biomarker. Cerebral Cortex. 2020;30(11):5844-5862.

20. Truelove-Hill M, Erus G, Bashyam V, et al. A multidimensional neural maturation index reveals reproducible developmental patterns in children and adolescents. Journal of Neuroscience. 2020;40(6):1265-1275.

21. Tønnesen S, Kaufmann T, de Lange A-MG, et al. Brain age prediction reveals aberrant brain white matter in schizophrenia and bipolar disorder: A multisample diffusion tensor imaging study. Biological Psychiatry: Cognitive Neuroscience and Neuroimaging. 2020;5(12):1095-1103.

22. Chen C-L, Hsu Y-C, Yang L-Y, et al. Generalization of diffusion magnetic resonance imaging–based brain age prediction model through transfer learning. NeuroImage. 2020;217:116831.

23. Bashyam VM, Erus G, Doshi J, et al. MRI signatures of brain age and disease over the lifespan based on a deep brain network and 14 468 individuals worldwide. Brain. 2020;143(7):2312-2324.

24. Shahab S, Mulsant BH, Levesque ML, et al. Brain structure, cognition, and brain age in schizophrenia, bipolar disorder, and healthy controls. Neuropsychopharmacology. 2019;44(5):898-906.

25. Kaufmann T, van der Meer D, Doan NT, et al. Common brain disorders are associated with heritable patterns of apparent aging of the brain. Nature neuroscience. 2019;22(10):1617-1623.

26. Hajek T, Franke K, Kolenic M, et al. Brain age in early stages of bipolar disorders or schizophrenia. Schizophrenia bulletin. 2019;45(1):190-198.

27. Kolenic M, Franke K, Hlinka J, et al. Obesity, dyslipidemia and brain age in first-episode psychosis. Journal of psychiatric research. 2018;99:151-158.

28. Chung Y, Addington J, Bearden CE, et al. Use of machine learning to determine deviance in neuroanatomical maturity associated with future psychosis in youths at clinically high risk. JAMA psychiatry. 2018;75(9):960-968.

29. Nenadić I, Dietzek M, Langbein K, et al. BrainAGE score indicates accelerated brain aging in schizophrenia, but not bipolar disorder. Psychiatry Research: Neuroimaging. 2017;266:86-89.

30. Schnack HG, Van Haren NE, Nieuwenhuis M, et al. Accelerated brain aging in schizophrenia: a longitudinal pattern recognition study. American Journal of Psychiatry. 2016;173(6):607-616.

31. Koutsouleris N, Davatzikos C, Borgwardt S, et al. Accelerated brain aging in schizophrenia and beyond: a neuroanatomical marker of psychiatric disorders. Schizophrenia bulletin. 2014;40(5):1140-1153.
